# 

**Published:** 1889-02

**Authors:** 


### Editorial, Etc.

---

UNIVERSITY OF MARYLAND DENTAL DEPARTMENT.—The Annual Commencement exercises of this Dental Department will be held at the Lyceum Theatre, Baltimore City, on the evening of March 13th, 1889.

The Address to the Graduating Class will be delivered by Dr. J. B. Patrick, of Charleston, South Carolina. The Class Address will be delivered by Hampton K. Smith, of S. C., a member of the Graduating Class. William L. Fish, of Newark, New Jersey, is the President of the Graduating Class.

On the day preceding the annual Commencement, March 12th, 1889, the "Class Contest" for prizes will take place in the Dental Infirmary of the University, beginning at 10 A. M. On the evening of Commencement day, March 13th, 1889, after the Commencement exercises, the Annual Meeting of the Alumni Association of the University of Maryland Dental Department will be held, followed by the Alumni Banquet. All friends of this Department are cordially invited to the Class Contest and Commencement exercises.

FERDINAND J. S. GORGAS, Dean.

### To Readers and Correspondents.

Communications intended for publication, and exchange journals and papers, should be addressed to the Editor of The American Journal of Dental Science, No. 845 N. Eutaw St. Baltimore, Md. All letters relating to the business of the Journal, or containing cash remittances, to be directed to Messrs. Snowden & Cowman, No. 9 W. Fayette Street, Baltimore, Md. Articles for publication should be received by the Editor at least two weeks previous to the first of the month on which the number in which it is designed for them to appear, is issued.

☞ The American Journal of Dental Science will contain fifty pages of reading matter in each number, making a volume of about Six Hundred pages,

EXCLUSIVE OF ADVERTISEMENTS.

